# Male and female meiotic behaviour of an intrachromosomal insertion determined by preimplantation genetic diagnosis

**DOI:** 10.1186/1755-8166-3-2

**Published:** 2010-02-08

**Authors:** Leoni Xanthopoulou, Anna Mantzouratou, Anastasia Mania, Suzanne Cawood, Alpesh Doshi, Domenico M Ranieri, Joy DA Delhanty

**Affiliations:** 1 UCL Centre for PGD, Institute for Women's Health, University College London, 86-96 Chenies Mews, London WC1E 6HX, UK; 2 Centre for Reproductive and Genetic Health, University College Hospital, The New Wing, Eastman Dental Hospital, London WC1X 8LD, UK; 3 IVF Hammersmith, Hammersmith Hospital, Du Cane Road, London W12 0HS, UK

## Abstract

**Background:**

Two related family members, a female and a male balanced carrier of an intrachromosomal insertion on chromosome 7 were referred to our centre for preimplantation genetic diagnosis. This presented a rare opportunity to investigate the behaviour of the insertion chromosome during meiosis in two related carriers. The aim of this study was to carry out a detailed genetic analysis of the preimplantation embryos that were generated from the three treatment cycles for the male and two for the female carrier.

Patients underwent *in vitro *fertilization and on day 3, 22 embryos from the female carrier and 19 embryos from the male carrier were biopsied and cells analysed by fluorescent in situ hybridization. Follow up analysis of 29 untransferred embryos was also performed for confirmation of the diagnosis and to obtain information on meiotic and mitotic outcome.

**Results:**

In this study, the female carrier produced more than twice as many chromosomally balanced embryos as the male (76.5% vs. 36%), and two pregnancies were achieved for her. Follow up analysis showed that the male carrier had produced more highly abnormal embryos than the female (25% and 15% respectively) and no pregnancies occurred for the male carrier and his partner.

**Conclusion:**

This study compares how an intrachromosomal insertion has behaved in the meiotic and preimplantation stages of development in sibling male and female carriers. It confirms that PGD is an appropriate treatment in such cases. Reasons for the differing outcome for the two carriers are discussed.

## Background

Intrachromosomal insertions are rare forms of chromosomal rearrangements -so far reported only in about 30 families [1] - that have a high impact on reproductive outcome for a carrier parent. They involve three breaks occurring in the same chromosome; the segment that is free intercalates into another part of the same chromosome, either within the same chromosome arm (within arm insertion) or into the other chromosome arm (between arm insertion). Moreover depending on the orientation of the inserted segment in relation to the centromere, insertions can be direct or inverted.

Balanced carriers of direct intrachromosomal insertions are phenotypically normal but are at risk of unbalanced meiotic segregation due to crossing over. The different segregation patters at meiosis can produce balanced or unbalanced gametes due to the deletion or duplication of the inserted segment, with the risk of live born unbalanced offspring varying from 15% to 50% [2,3]. Since there is no reciprocal segment involved in this type of structural abnormality, meiotic segregation alone may result in pure monosomy or trisomy for the inserted segment. Previous reports on intrachromosomal insertions involved the birth of individuals with abnormal phenotypes, however Farrell and Chow [4] reported a case of an intrachromosomal insertion on chromosome 7, which presented with recurrent pregnancy losses. The genetic risk depends on the chromosome involved in the abnormality and the position of the breakpoints, i.e. the length of the inserted segment and the centromeric segment. In this way the smaller the inserted segment, the more likely it is that it could be tolerated in the trisomic or monosomic state and lead to viable unbalanced offspring [5]. Moreover, the longer the centromeric segment in proportion to the length of the whole chromosome, the more chances there are for crossing over events to occur, leading to unbalanced recombinant chromosomes.

**Figure 1:**
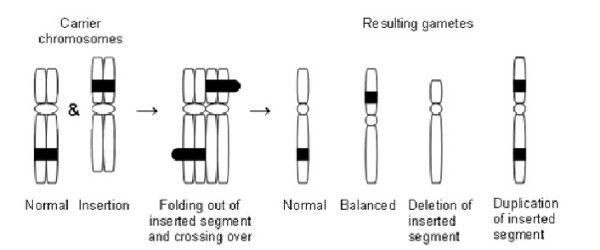
**Incomplete synapsis at meiosis in a balanced carrier of a direct between arm intrachromosomal insertion**.

In the case of a small inserted segment (less than 1% of haploid length - HAL) the most likely meiotic scenario is of incomplete synapsis [3]. In this situation, only the centromeric segment pairs and the inserted segment loops out, as shown in figure 1. An odd number of crossovers then produces a 1:1:1:1 ratio of gametes that are normal, duplicated or deleted for the insertion or balanced carriers, i.e. with a theoretical 50% risk of abnormality. Duplication of a small inserted segment is more likely to be tolerated, but the exact outcome will vary depending upon the genetic background, however deletion of the segment is likely to lead to miscarriage.

The high reproductive risks associated with chromosomal insertions makes them good candidates for preimplantation genetic diagnosis (PGD), an alternative to prenatal diagnosis, carried out on embryos created by in vitro fertilisation (IVF) when they are three days old [6]. PGD involves the removal of one or two single blastomeres from day 3 cleavage stage embryos [7]. The cells are subsequently tested using the polymerase chain reaction (PCR) for single gene disorders [8] or fluorescent in situ hybridization (FISH) for chromosomal abnormalities [9], thereby allowing only normal or balanced embryos to be transferred, with the aim of establishing an unaffected pregnancy.

To date there has only been a single publication devoted solely to PGD for an insertion; this was for a case of an interchromosomal insertion [10], however there were no follow up studies on the abnormal embryos. Here we report on the outcome of several PGD cycles with detailed follow-up analysis, carried out for a brother and a sister that are both carriers of a direct between arm intrachromosomal insertion affecting chromosome 7 and discuss possible reasons for the differing outcome for the couples involved.

## Patients and Consent

Two related family members, a brother and a sister, both balanced carriers of a direct between-arm intrachromosomal insertion on chromosome 7 were referred for PGD. Patient details and information on their reproductive histories are given in table 1. The male carrier and his partner are described as sub-fertile due to a period of infertility and a falling sperm count, such that intracytoplasmic sperm injection (ICSI) was required for the second and third cycles of PGD.

**Table 1:** Patients' reproductive histories prior to PGD.

Case	Fertility status	Maternal age	Normal live births	Chromosomally abnormal live births	Previous PND and TOP or miscarriages
Female carrier	Fertile	1^st ^cycle: 33	1	0	1 m/c,
		2^nd ^cycle: 39			1 × TOP

Male carrier	Subfertility, falling sperm count	1^st ^cycle: 37	0	2	0
		2^nd ^cycle: 37			
		3^rd ^cycle: 38			

Key: PND: Prenatal diagnosis, TOP: Termination of pregnancy, m/c: miscarriage

The male carrier has a mildly affected 16 year old son with an unbalanced form of the insertion (duplication of the inserted segment) and also had a daughter with duplication of the inserted segment that died aged 10 months. A third child with normal chromosomes was born from a natural conception shortly after their PGD treatment finished. The female carrier had a normal child, a miscarriage at 11 weeks and a termination of an unbalanced pregnancy at 17 weeks (duplication of the inserted segment).

Both individuals and their partners were karyotyped by clinical cytogeneticists prior to the onset of treatment. The karyotypes of the balanced carriers were 46, XY, dir ins [7](p22q32q31.1) and 46, XX, dir ins [7](p22q32q31.1).

Treatment for these couples was approved by the UK Human Fertilization and Embryology Authority (HFEA), and written informed consent was obtained for all the procedures; the patients had also attended several thorough IVF/PGD consultations which covered all aspects of the procedures involved. Written consent was obtained from the patients for publication of this case report and accompanying images. A copy of the written consent is available for review by the Editor-in-Chief of this journal.

## Methods

**Figure 2:**
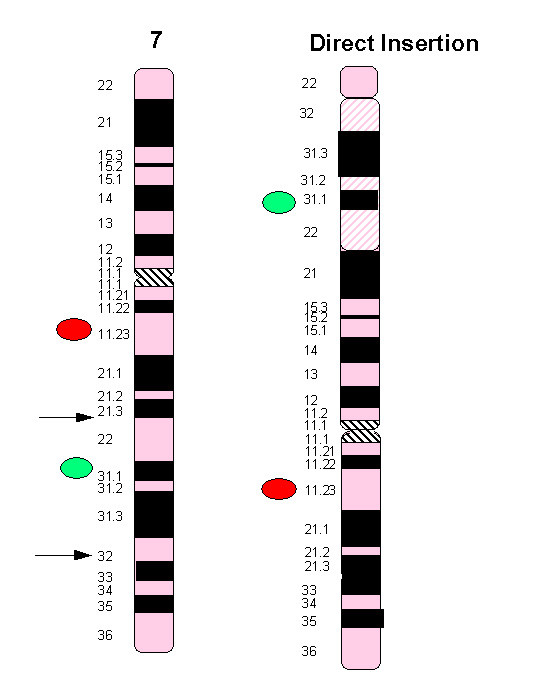
**Ideogram and FISH probe strategy used on embryonic nuclei in PGD**. The Williams dual band probe was used, which binds to a control site on 7q11.23 in Spectrum Orange and to a site included within the inserted segment on 7q31 in Spectrum Green. The centromeric probe for chromosome 15 in Spectrum Aqua was also used.

Based on the cytogenetic reports for the two carriers, a probe strategy was decided, shown in Figure 2. The FISH probe strategy involved the use of the Williams dual band probe that binds to a control site on 7q11.23 in Spectrum Orange and to a site included within the inserted segment in Spectrum Green, together with the centromeric probe for chromosome 15 in Spectrum Aqua in order to monitor the ploidy status (all probes from Abbott, UK). In certain cycles additional probes were used for chromosomes 13, 18, 21 for an aneuploidy screen, but these results are not presented here.

Slide preparation for FISH and standard methods of processing are as described in Simopoulou *et al*. [6]. The efficiency of the protocol was calculated by scoring the signals in 200 interphase nuclei from each parental sample, based on the scoring criteria of Hopman *et al*. [11], using an epifluorescence Olympus microscope (Olympus BX 40) paired with a Photometrics cooled CCD camera utilising the Smartcapture software (Digital Scientific, UK).

**Figure 3:**
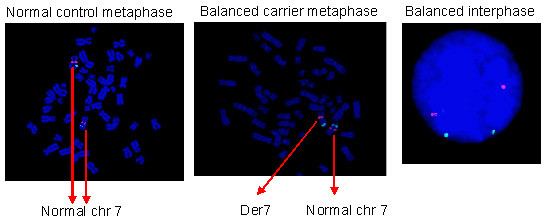
**Development of the molecular cytogenetic protocol on parental lymphocytes**. A metaphase from the normal parent and a metaphase from the carrier parent are shown, as well as a balanced interphase nulceus. Note that here only the Williams dual band probe is shown (7q11.23 in Spectrum Orange and 7q31 in Spectrum Green).

**Figure 4:**
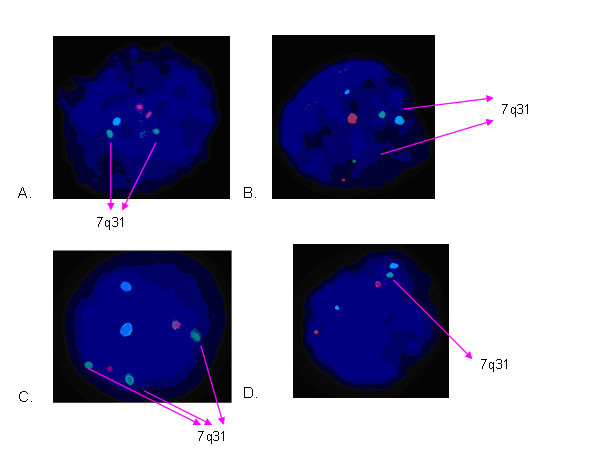
**FISH results on biopsied interphase blastomere (embryonic) nuclei**. The Williams dual band probe (7q11.23 in Spectrum Orange, 7q31 in Spectrum Green) and the centromeric probe for chromosome 15 in Spectrum Aqua were used. A and B are examples of balanced or normal nuclei, whereas C is unbalanced due to the duplication of the Inserted segment and D is unbalanced due to the deletion of the inserted segment.

Once the work up was completed (figure 3) the patients underwent IVF stimulation, vaginal oocyte collection, insemination, embryo biopsy and blastomere spreading as described in Mantzouratou *et al*. [12]. From the majority of embryos 2 blastomeres were taken and analysed using the optimised FISH protocol (figure 4). The signals were scored under the microscope and all scoring decisions were made by at least two observers. The embryonic nuclei obtained for PGD were in the interphase state, so it was not possible to distinguish between balanced carriers and normal embryos [13].

Mosaicism was recorded when nuclei with differing number of probe signals were found in the same embryo. In the most extreme form, chaotic mosaicism, signal numbers vary randomly from cell to cell with no discernable mechanism. Embryos may be fully chaotic, in which case, the meiotic segregation cannot always be determined, or partially, so, with a core of cells with balanced or unbalanced chromosomes.

Two PGD cycles for the female carrier (overall 22 embryos biopsied) and three PGD cycles for the male carrier (overall 19 embryos biopsied) are described here. The first PGD treatment cycle of the female carrier was briefly reported as patient F in Simopoulou *et al*. [6]. Follow up analysis of 29 untransferred embryos (13 from the female carrier and 16 from the male carrier) on day 5/6 of embryo development was also performed using the same protocol used for the analysis of the biopsied cells in order to confirm the diagnosis and investigate the status of the insertion chromosome in these embryos. These results are are presented here.

## Results

Table 2 summarises the details of the 5 PGD treatment cycles for the two couples. From the two PGD cycles of the female carrier, a total of 29 oocytes were collected and inseminated, resulting in the biopsy of 22 embryos, whereas from the 3 PGD cycles of the male carrier, 28 oocytes were collected, 27 were fertilised, resulting in the biopsy of 19 embryos.

**Table 2:** Summary of the PGD treatment cycles.

Case	Cycle	Oocytes collected	Oocytes fertilised	Embryos biopsied
**Female carrier**	1	16	16	11

	2	13	13	11

	**Total**	29	29	22

**Male carrier**	1	5	5	5

	2*	15	15	9

	3*	8	7	5

	**Total**	28	27	19

Two PGD cycles carried out for the female carrier of the intrachromosomal insertion and the 3 PGD cycles carried out for the male carrier of the insertion.

* ICSI (Intracytoplasmic Sperm Injection)

The biopsy results for these embryos are listed on Additional file 1. The female carrier had overall 11 (52.4%) balanced embryos, 7 unbalanced embryos due to deletion or duplication and 1 embryo with no results, whereas the male carrier had overall 6 (35.3%) balanced embryos, 7 unbalanced embryos and 2 embryos with no result. Hybridization failure, loss of the biopsied blastomere as well as background noise may have resulted in inconclusive or no result in certain cases. Other abnormalities made up the total of abnormal embryos to 10 (48%) in the female and 11 (65%) in the male.

A pregnancy and the birth of a healthy girl was achieved after the 1^st ^PGD cycle for the female carrier, whereas after the second cycle a pregnancy was established but was ectopic. For the male carrier no pregnancies were established from the first 2 PGD cycles and there was no embryo transfer from the 3^rd ^cycle.

There were 13 untransferred embryos from the female carrier and 16 from the male carrier, which were followed up. Detailed analysis of those is presented in table 3, where embryos are classified according to their chromosomal status. The meiotic segregation patterns deduced from the biopsied cells and the overall data including that from the follow up data on the untransferred embryos are also presented in Additional File 1 and  Table 4.

**Table 3:** Summary of embryo classification based on the biopsy and follow up results.

Embryo characterization	Number of embryos (Female carrier)	Number of embryos (Male carrier)
Balanced	10 (50%)	3 (19%)

Unbalanced	2	2

Balanced/chaotic mosaic	3	2

Unbalanced/chaotic mosaic	2 (10%)	5 (31.3%)

Fully chaotic	3 (15%)	4 (25%)

No result	2	3

**Total**	**22**	**19**

**Table 4:** Overall meiotic segregation patterns in the embryos based on the biopsy and follow up results.

Meiotic segregation		Number of embryos (Female carrier)	Number of embryios (Male carrier)
**Balanced**		13 (76.5%)	5 (36%)

**Unbalanced**	Duplication 7q31	3	5

	Deletion 7q31	1	4

	Total unbalanced	4 (23.5%)	9 (64%)

**Unknown**		5	5

**Total**		**22**	**19**

A summary of these results, comparing the results from the 2 PGD cycles of the female carrier with the results of the 3 PGD cycles of the male carrier is presented in Additional file 1, table S3. Unbalanced meiotic segregation was more than twice as frequent in the male carrier compared to the female sibling and, conversely, the balanced segregation type was twice as frequent in her gametes compared with his sperm.

## Discussion

In the preimplantation stages of development it is possible to see the products of all the different segregation patterns of the insertion chromosome, which might not be viable in later stages of development. This study therefore provides us with a unique insight into the meiotic segregation of the insertion chromosome at oogenesis and spermatogenesis in balanced female and male carrier siblings of the same insertion.

As mentioned earlier, in the literature to date there is only one publication devoted solely to PGD for an insertion and that was of the interchromosomal type [10]. In that study however no follow up analysis was carried out so there was no confirmation of the meiotic errors that were proposed. These could have come about from postzygotic mitotic errors and be the result of mosaicism. Our study however includes follow up analysis of the untransferred embryos, allowing us therefore to analyse the meiotic behaviour of the intrachromosomal insertion chromosome deduced from the preimplantation embryos.

Postnatal studies report no difference in fertility between male and female interchromosomal insertion carriers [14] and estimate the risk of having an abnormal child as being close to 32% for male carriers, compared to 36% for female carriers [3], whereas no reports have been made for intrachromosomal insertions.

In this study however, it is clear that apart from the fact that the female carrier already had a normal child at the time of referral (table 1), she produced twice as many balanced embryos as did the male, and pregnancies were also achieved for the female in both PGD cycles (table 4).

The two children of the male carrier that had duplication of the inserted segment are proof that imbalance resulting from unfavourable meiotic segregation from this intrachromosomal insertion can be viable. However there are no live offspring with the deleted segment. The inserted segment in this family is 0.46% HAL, i.e. smaller than 1% HAL, which means that incomplete synapsis at meiosis is the most likely scenario whereby pairing without crossing over in the centromeric segment will not lead to abnormal gametes. The short inserted segment reduces the chance of crossing over within the inserted region but the large centromeric segment increases the chance of crossing over in that segment leading to a high risk of abnormal gametes. If one or an odd number of cross overs occurs then a 1:1:1:1 ratio of normal:deleted:duplicated:balanced gametes will be produced. Indeed in our results there is evidence for all the different segregation patterns both in the biopsy results on day 3 and in the follow up results on day 5 (Additional file 1 and table 4). The results however are different for the female and the male carrier, i.e. the meiotic segregation patterns deduced for the same intrachromosomal insertion differ according to the sex of the carrier parent. Since crossing over is higher in human females than males [15], it is possible that in the female carrier double crossover in the centromeric segment produces more balanced gametes. It could be therefore the difference in the rate of recombination between the male and the female that accounts for the difference in the behaviour of the insertion chromosome at meiosis.

The most common meiotic segregation pattern was balanced segregation in the female carrier (76.5% compared to 36% in the male). The majority of the embryos of the male carrier were the result of unbalanced segregation (64% compared to 23.5% for the female, table 4). There was therefore a different preferential segregation pattern in the male and female partner, based on the available data. In the unbalanced embryos from both carriers, the most common segregation pattern was the duplication of the inserted segment (table 4).

Tease and Hulten [16] argue that the fact that chromosomes are packaged differently in oocytes than in sperm accounts for the different rates of recombination in the male and female germ cells. More specifically in a previous study Tease [17] analysed the segregation patterns seen in male and female mice that carried the same reciprocal translocations and noted that in females there was a higher frequency of chiasmata and that chiasmata were often interstitial, concluding that in mice the localization and the number of chiasmata formed differs between the sexes.

Apart from the recombination rates being sex specific, other mechanisms have been proposed, to explain these differences observed between the sexes Aside from the frequency and position of the chiasmata, chromosome pairing, the initiation of synapsis as well as the quality of synapsis are also different between males and females [18], whereas the genetic background is also thought to influence chromosome behaviour at meiosis [17]. Since our two insertion carriers are siblings they are expected to share 50% of their genome. In a study on the meiotic segregation of the same Robertsonian translocation in female and male pigs that were related, i.e. of similar genetic background, Pinton *et al*. [19] report that the rate of balanced gametes was lower in the female carriers. Although meiotic errors in the males may cause spermatogenesis to arrest, female meiosis is more prone to errors since the meiotic checkpoints are less efficient Hunt and Hassold [20]. Pinton *et al*. [19] propose that a similar aetiology could be behind their findings. Other studies such as that by Underkoffler *et al*. [21] report contrary findings, i.e. that in mouse carriers of Robertsonian translocations, female carriers produce more normal gametes than male carriers, due to the preferential segregation of the derivative chromosomes to the first polar body. This scenario of the oocyte correcting itself by including the abnormal chromatids in one of the polar bodies could also be in play in humans.

In human carriers of reciprocal translocations Ogilvie and Scriven [22] report that for female carriers balanced segregation was seen in 60% of the embryos compared to 43% for the male carriers. It would therefore appear that female carriers are more likely to produce a higher rate of balanced gametes, therefore having a higher chance of a balanced good quality embryo during PGD. Regardless of the mechanism involved, it is clear from the literature as well as from our results that the sex of the carrier parent has an effect on chromosome segregation.

In our family, the progressively worsening sperm count for the male carrier could have had an adverse effect on embryonic development, particularly that due to mosaicism. In humans the male contributes the centrosome [23] which could function less efficiently in poor quality sperm. An abnormal number of centrosomes as well as centrosome dysfunction would lead to abnormal cell division [23,24]. Indeed Magli *et al*. [25] looked into embryos from couples where there was male factor infertility and the female partner was younger than 36 years of age, and reported a higher incidence of postmeiotic abnormalities, particularly chaotic mosaics, in the couples where the male factor was more severe. Mantzouratou et al. [26] also reported that couples in need of ICSI had more chaotic errors in their embryos. Consequently for the correct chromosome segregation into the daughter cells, sperm integrity is crucial, as this will determine centrosome function, which is necessary for correctly setting up the first mitotic cleavage divisions.

Follow up analysis on the 29 untransferred embryos, provided a very rare opportunity to monitor the mitotic behaviour for the insertion chromosome as well. Postzygotic errors were widespread in all embryos studied resulting in a high degree of mosaicism. Human preimplantation embryos generated though IVF show a high level of mosaicism [27-30] and it has also been well established that the frequency of aneuploidies is much higher than originally anticipated [31,32]. Depending on the proportion of abnormal cells present and on their distribution, mosaicism can often be a minor problem for the development of preimplantation embryos, when for instance they are not involved in the formation of the inner cell mass [28,33]. However widespread mosaicism will affect embryo development. Mosaicism has been reported not only in arrested or fragmented embryos but also in normally developing embryos of good quality from fertile patients [28] and has been observed in the embryos of both young and older women [26,34]. Different factors are thought to be involved in the formation of mosaic embryos, such as the ovarian stimulation protocol used or the embryo culture conditions, as well as the fact that cell-cycle checkpoints are not fully functional during the early stages of preimplantation embryo development [35]. Furthermore a study on fertile patients, pointed towards the predisposition of certain couples to the production of chaotic embryos, i.e. embryos showing an extreme form of mosaicism, whereby every cell has a different chromosomal complement [28]. Carriers of chromosomal abnormalities presenting for PGD have often been reported to also suffer from this tendency to produce highly mosaic embryos [36].

For the female carrier, after the first treatment cycle, (apart from the five frozen embryos with balanced chromosomes 7 which were not available for follow up), there was one uniformly balanced embryo, two uniformly unbalanced embryos and one chaotic embryo. From her second cycle, however there were two unbalanced/chaotic embryos, three balanced/chaotic embryos, two chaotic embryos and two embryos with a no result. Because there was a gap of several years between those two treatment cycles, increased maternal age (33 years in the first cycle versus 39 years in the second cycle) could be a factor for the diploid/aneuploid mosaicism seen, due to mitotic non-disjunction which is related to the maternal age [37,38].

Overall the male carrier had produced more fully chaotic embryos than the female (25% and 15% respectively, additional file 1 and table 3), similarly the frequency of the unbalanced/chaotic mosaic embryos was also higher in the male compared to the female carrier (31.3% compared to 10%, table 3). Simopoulou *et al*. [6] argue that some carriers of chromosomal abnormalities that resort to PGD are at a risk not only of an unfavourable meiotic segregation but also of uncontrolled, chaotic postzygotic divisions, producing highly mosaic embryos [39]. This patient related predisposition towards the production of chaotic embryos has been established [26,28] and in this way, extreme chaotic mosaicism seen in most embryos in the male carrier might be related to the sub fertility of this couple.

## Conclusion

This is a unique study that shows how an intrachromosomal insertion has behaved in the early preimplantation stages of development in offspring from sibling male and female carriers, both at the meiotic and at the post-zygotic mitotic stage. It also proves that PGD is an appropriate treatment for an intrachromosomal insertion due to the high risk of unbalanced meiotic segregation.

## Competing interests

The authors declare that they have no competing interests.

## Authors' contributions

LX was the main author and was responsible for all the preliminary work up and optimization of the protocol as well as all data collection. and analysis. AM and AM were involved in preliminary work, single blastomere spreading and biopsy data collection. SC, AD were the main embryologists in the cases and DMR was the main physician As the director of the PGD Centre, JDAD was involved in all aspects of these PGD cycles presented here and editing the manuscript. All authors read and approved the final manuscript.

## Supplementary Material

Additional file 1 **Detailed biopsy results and follow up analysis of the untransferred embryos from both carriers**. This table outlines the embryo classification and meiotic segregation pattern seen. (Balanced refers to the biopsy results for chromosome 7, indicating embryos normal for chromosome 7 or balanced carrier of the insertion)
Click here for file
