# Supplementary material for: Male and female meiotic behaviour of an intrachromosomal insertion determined by preimplantation genetic diagnosis
Source: Mol Cytogenet. 2010 Feb 8;3:2. doi: 10.1186/1755-8166-3-2 (PMC2830219; doi:10.1186/1755-8166-3-2)
Supplement: Supplementary file 1 [file 1755-8166-3-2-S1.DOC]

Couple	Cycle	Embryos biopsied	Number of embryos transferred	Outcome	Follow up analysis on day 5/6	Meiotic segregation: number of embryos with evidence for that type of segregation based on the follow up results
Female carrier	1	11<br>(Normal/balanced: 7<br>Abnormal: 3<br>No result: 1)	2 (another 5 balanced frozen)	Normal live birth	Balanced: 1<br>Unbalanced: 2<br>Chaotic: 1	Balanced: 1<br>Deletion 7q31: 1<br>Duplication 7q31: 1<br>Unknown: 1
	2	11<br>(Normal/balanced: 4<br>Abnormal: 7<br>No result: 0)	2	Pregnancy but ectopic	Unbalanced/chaotic mosaic: 2<br>Balanced/chaotic mosaic: 3<br>Fully chaotic: 2<br>No result: 2 (1 degenerate embryo)	Balanced: 3<br>Duplication 7q31: 2<br>Unknown: 4
Male carrier	1 (IVF)	5<br>(Normal/balanced: 4<br>Abnormal: 1<br>No result: 0)	2	No pregnancy	Balanced/chaotic mosaic: 1<br>Chaotic: 1<br>Unbalanced/Chaotic mosaic: 1	Duplication of 7q11.23: 1<br>Balanced: 1<br>Unknown: 1
	2 (ICSI)	9<br>(Normal/balanced: 2<br>Abnormal: 6<br>No result: 1)	1	No pregnancy	Unbalanced/chaotic mosaic: 3<br>Balanced/unbalanced mosaic: 1<br>Unbalanced mosaic: 1<br>Fully chaotic: 3	Deletion 7q31: 3<br>Duplication 7q31: 3<br>Balanced: 1<br>Unknown: 1
	3 (ICSI)	5<br>(Normal/balanced: 0<br>Abnormal: 4<br>No result: 1)	0	-<br>(No transfer)	Unbalanced mosaic: 1<br>Unbalanced/chaotic mosaic: 1<br>No result: 3 (degenerate embryos)	Deletion 7q31: 1<br>Duplication 7q31: 1<br>Unknown: 3
